# An insight on gender‐based violence

**DOI:** 10.1002/hsr2.1815

**Published:** 2024-01-11

**Authors:** Khushi Ochani, Amna Siddiqui, Sidhant Ochani

**Affiliations:** ^1^ Department of Dentistry Dow University of Health Sciences Karachi Pakistan; ^2^ Department of Medicine Karachi Medical and Dental College Karachi Pakistan; ^3^ Department of Medicine Khairpur Medical College Khairpur Mir's Pakistan

Respected Editor,

Gender‐based violence (GBV) is a global concern for health and human rights, defined as any harmful act targeted at an individual or group based on their gender. It is deeply rooted in gender inequality, the misuse of power, and detrimental norms.^1^ This term encompasses individuals from all backgrounds, irrespective of their socioeconomic status. Although not always perpetrated by men against women, which is the most common global manifestation, it stems from imbalanced power dynamics between men and women and factors such as illiteracy, poverty, and low social standing. Risk factors linked to engaging in violence include a history of alcohol abuse and family violence, poor mental health, low education and socioeconomic status, borderline personality disorders, and emotional insecurities.^2^

When directed at women, GBV poses a significant barrier to reproductive health, resulting in increased maternal mortality, unsafe abortions, restricted access to prenatal care, insufficient maternal weight gain, and an increased likelihood of adolescent pregnancies. This can lead to unintended pregnancies, complications, sexually transmitted infections such as human immunodeficiency virus, depression, posttraumatic stress disorder, and, in the most severe cases, fatalities.^3^ Despite its global impact, GBV is not acknowledged as a public health issue, thus it is necessary to understand the impact of GBV on the health and well‐being of women and children (https://www.who.int/news/item/25-11-2021-gender-based-violence-is-a-public-health-issue-using-a-health-systems-approach).^4^ Hence, our letter addresses the crucial issue of GBV worldwide and proposes implementations that can aid in curtailing the same.

GBV transcends age, religion, classes, and national boundaries, manifesting itself in various contexts such as the family, workplace, public spaces, communities, states, and even during pregnancy or times of conflict.^4^ Domestic violence against women or violence that occurs between intimate partners is the most prevalent form of GBV.^1^ Studies reveal that one in seven homicides worldwide is perpetrated by an intimate partner. While men are at greater risk of homicide overall, the proportion of murdered women killed by an intimate partner is six times higher than for murdered men.^5^ GBV is pervasive across many Asian‐Pacific countries, yet it often remains a topic shrouded in social silence. However, there is a growing acknowledgment of GBV as a significant public health concern in the region. Research conducted in India indicates a connection between experiencing physical violence, a reduced inclination to adopt contraception, and an increased likelihood of unwanted pregnancies.^6^ Similarly, studies in various countries, such as the Maldives and Pakistan, have identified that instances of physical abuse are linked to higher rates of adverse pregnancy outcomes, including miscarriages, late pregnancy bleeding, premature labor or delivery, stillbirths, abortions, and delayed initiation of prenatal care (https://arrow.org.my/wp-content/uploads/2015/04/AFC-Vol.17-No.2-2011_Gender-based-Violence-and-Health-Sector.pdf). Notably, during the pandemic, GBV experienced an alarming surge, China witnessed a tripling of cases and helpline calls increasing by 20‐90% in countries like Colombia, Mexico, Australia, Cyprus, and the United States.^7^ A comprehensive study that investigates the impact of the COVID‐19‐related lockdown on the mental health of Tunisian women and the occurrence of GBV, this research covers all regions of Tunisia and involves 751 participants. The findings reveal that more than half of the participants (57.3%) reported extremely severe distress symptoms. Additionally, women who encountered abuse faced an increased risk of psychological violence, evident in 96% of cases.^8^ Moreover, recent data have shown that the prevalence of sexually transmitted diseases among women who have experienced violence is twice as high as in women who have not.^9^ The countless statistics available online shed light on the pervasive nature of GBV, necessitating a global response with stringent measures in place to combat this issue effectively.

To tackle GBV, various essential measures can be implemented. In situations where services to address violence against women are limited, establishing a 24‐hour emergency response, including shelter safety planning and legal aid, would help ensure the safety of survivors in immediate danger. Concurrently, awareness campaigns should be designed considering the cultural and social nuances of specific regions, such as developing countries. It is imperative to enhance healthcare workers’ understanding of GBV through targeted training programs that consider local customs. Additionally, strict laws must be enacted to serve as deterrents, punishing abusers and setting examples for society. Further, regular screenings are vital for early detection of GBV cases, while accounting for cultural norms and the social dynamics between healthcare providers and patients. Importantly, promoting women's literacy and financial independence is of utmost importance, enabling them to challenge gender roles and break free from abusive situations. Considerably, strengthening social support networks through community‐based programs is also crucial, taking into consideration the unique challenges faced by different communities (https://www.concern.net/news/solutions-to-gender-based-violence). Lastly, behavioral interventions addressing the root causes of GBV, such as harmful gender norms, will contribute to fostering healthy relationships and achieving equality. However, it is essential to recognize that healthcare screening practices may vary across regions, as cultural factors, such as husbands accompanying wives to medical appointments or social ties between healthcare providers and patients in rural areas, can impact their effectiveness. Therefore, it is essential to adapt all interventions to local communities and conditions, drawing on the wisdom and experiences shared by local survivors of GBV. Hence, by incorporating these localized perspectives, the fight against GBV can make significant progress on a global scale.^10^

## AUTHOR CONTRIBUTIONS

**Khushi Ochani**: Writing—original draft, writing—review and editing. **Amna Siddiqui**: Writing—original draft, writing—review and editing. **Sidhant Ochani**: Resources, writing—original draft, writing—review and editing.

## CONFLICT OF INTEREST STATEMENT

The authors declare no conflict of interest.

## ETHICS STATEMENT

Not applicable.

## Data Availability

Data available on request from the authors.
